# Supplementary material for: A quantum circuit simulator and its applications on Sunway TaihuLight supercomputer
Source: Sci Rep. 2021 Jan 11;11:355. doi: 10.1038/s41598-020-79777-y (PMC7801639; doi:10.1038/s41598-020-79777-y)
Supplement: Supplementary file 1 — Supplementary Information. [file 41598_2020_79777_MOESM1_ESM.pdf]

Supplementary material for: A quantum circuit simulator and its applications on Sunway TaihuLight supercomputer

Zhimin Wang[1], Zhaoyun Chen[2,3], Shengbin Wang[1], Wendong Li[1], Yongjian Gu[1*], Guoping Guo[2,3*], Zhiqiang Wei[1,4*]

[1] College of Information Science and Engineering, Ocean University of China, Qingdao 266100, China
[2] CAS Key Laboratory of Quantum Information, University of Science and Technology of China, Hefei 230026, China
[3] Origin Quantum Computing Company Limited, Hefei 230026, China
[4] High Performance Computing Center, Pilot National Laboratory for Marine Science and Technology (Qingdao), Qingdao 266100, China
[*] Correspondence author: yjgu@ouc.edu.cn (Y. Gu); gpguo@ustc.edu.cn (G. Guo); weizhiqiang@ouc.edu.cn (Z. Wei)

In this supplementary material, a description of the instruction set of our simulator is given in Section S-1. Section S-2 shows an example of the input and output to illustrate the way of running the simulator.

S-1 Instruction set

Below each instruction is shown with its name, a brief description of its function, the syntax, and an argument about the syntax.

QINIT

Description: specify the number of qubits of the circuit which are initialized as |0>.
Syntax: QINIT *n*
Argument: *n* is the number of qubits of the quantum circuit.

CREG

Description: allocate classical registers for storing the measurement results.
Syntax: CREG *m*
Argument: *m* is the number of classical registers.

H/X/Y/Z/S/T gate

Description: perform Hadamard/Pauli-X/Pauli-Y/Pauli-Z/phase/T operations.
Syntax: H/X/Y/Z/S/T *i*
Argument: *i* represents a certain qubit ranging from 0 to *n*-1.

Operation: $H = \frac{\sqrt{2}}{2}\begin{bmatrix} 1 & 1 \\ 1 & -1 \end{bmatrix}, X = \begin{bmatrix} 0 & 1 \\ 1 & 0 \end{bmatrix}, Y = \begin{bmatrix} 0 & -i \\ i & 0 \end{bmatrix}, Z = \begin{bmatrix} 1 & 0 \\ 0 & -1 \end{bmatrix}, S = \begin{bmatrix} 1 & 0 \\ 0 & i \end{bmatrix}, T = \begin{bmatrix} 1 & 0 \\ 0 & e^{i\pi/4} \end{bmatrix}.$

RX/RY/RZ gates

Description: rotate the qubit about x-/y-/z-axis.
Syntax: RX/RY/ RZ *i*, "θ"
Argument: *i* represents a certain qubit from 0 to *n*-1 and θ is the rotation angle in radian.

Operation: $$RX(\theta)=\begin{bmatrix}\cos\frac{\theta}{2} & -i\sin\frac{\theta}{2}\\ -i\sin\frac{\theta}{2} & \cos\frac{\theta}{2}\end{bmatrix}, RY(\theta)=\begin{bmatrix}\cos\frac{\theta}{2} & -\sin\frac{\theta}{2}\\ \sin\frac{\theta}{2} & \cos\frac{\theta}{2}\end{bmatrix}, RZ(\theta)=\begin{bmatrix}e^{-i\theta/2} & 0\\ 0 & e^{i\theta/2}\end{bmatrix}.$$

U4 gates

Description: perform arbitrary sing-qubit gate.
Syntax: U4 *i*, "u_0, u_1, u_2, u_3"
U4 *i*, "α, β, γ, δ"
Argument: *i* represents a certain qubit from 0 to *n*-1. The u_0, u_1, u_2, u_3 or α, β, γ, δ are the elements or angles in radian in the matrix of the operator.

Operation: $$U4=\begin{bmatrix}u_0 & u_1\\ u_2 & u_3\end{bmatrix},\ U4=\begin{bmatrix}e^{i(\alpha-\frac{\beta}{2}-\frac{\delta}{2})}\cos\frac{\gamma}{2} & -e^{i(\alpha-\frac{\beta}{2}+\frac{\delta}{2})}\sin\frac{\gamma}{2}\\ e^{i(\alpha+\frac{\beta}{2}-\frac{\delta}{2})}\sin\frac{\gamma}{2} & e^{i(\alpha+\frac{\beta}{2}+\frac{\delta}{2})}\cos\frac{\gamma}{2}\end{bmatrix}.$$

CNOT/CZ/CR gate

Description: perform Pauli-X/Pauli-Z/Rotation-about-z-axis on the target qubit when the control qubit is |1>.
Syntax: CNOT/CZ *i*, *j*
CR *i*, *j*, "θ"
Argument: *i* and *j* represent two different qubits ranging from 0 to *n*-1, where *i* is the control qubit and *j* the target qubit. The θ is rotation angle in radian.

Operation: $$CNOT=\begin{bmatrix}1&0&0&0\\0&1&0&0\\0&0&0&1\\0&0&1&0\end{bmatrix},\ CZ=\begin{bmatrix}1&0&0&0\\0&1&0&0\\0&0&1&0\\0&0&0&-1\end{bmatrix},\ CR=\begin{bmatrix}1&0&0&0\\0&1&0&0\\0&0&1&0\\0&0&0&e^{i\theta}\end{bmatrix}.$$

SWAP/iSWAP gate

Description: swap the states of two qubits/swap the states of two qubits and add a $\pi/2$ phase.
Syntax: SWAP/ISWAP *i*, *j*
Argument: *i* and *j* represent two different qubits ranging from 0 to *n*-1.

Operation: $$SWAP=\begin{bmatrix}1&0&0&0\\0&0&1&0\\0&1&0&0\\0&0&0&1\end{bmatrix},\ iSWAP=\begin{bmatrix}1&0&0&0\\0&0&-i&0\\0&-i&0&0\\0&0&0&1\end{bmatrix}.$$

TOFFOLI gate

Description: perform NOT operation on the target qubit when the two control qubits are all in |1>.

Syntax: TOFFOLI *i*, *j*, *k*

Argument: *i*, *j* and *k* represent three different qubits ranging from 0 to *n*-1, where *i*, *j* are the control qubits and *k* the target qubit.

Operation: $$TOFFOLI = \begin{bmatrix} 1 & 0 & 0 & 0 & 0 & 0 & 0 & 0 \\ 0 & 1 & 0 & 0 & 0 & 0 & 0 & 0 \\ 0 & 0 & 1 & 0 & 0 & 0 & 0 & 0 \\ 0 & 0 & 0 & 1 & 0 & 0 & 0 & 0 \\ 0 & 0 & 0 & 0 & 1 & 0 & 0 & 0 \\ 0 & 0 & 0 & 0 & 0 & 1 & 0 & 0 \\ 0 & 0 & 0 & 0 & 0 & 0 & 0 & 1 \\ 0 & 0 & 0 & 0 & 0 & 0 & 1 & 0 \end{bmatrix}.$$

DAGGER & ENDDAGGER

Description: perform the inverse operation of a group of gates in the circuit.

Syntax: DAGGER

M

ENDDAGGER

Argument: DAGGER is the starting mark and ENDDAGGER is the terminating mark. Between them, the group of gates *M* is converted to its transpose conjugate $M^{\dagger}$ to be implemented. This instruction could be used in the nested way.

CONTROL & ENDCONTROL

Description: perform the control operation for a group of gates in the circuit.

Syntax: CONTROL *i*

M

ENDCONTROL *i*

Argument: CONTROL is the starting mark and END CONTROL is the terminating mark. Between them, the group of gates *M* is implemented controlled by the qubit *i*. This instruction could be used in the nested way.

MEASURE

Description: perform partial measurement on certain qubits and normalize the left quantum states to move on evolving.

Syntax: MEASURE *i*, $*j*

Argument: *i* represents a certain qubit ranging from 0 to *n*-1, and *j* behind $ represents a classical register specified by CREG. The measurement results are stored in the classical registers.

PMEASURE

Description: calculate the probability of the states in the space spanned by the specified qubits. No quantum states change in this measurement.

Syntax: PMEASURE $i, j, \ldots, k$

Argument: i to k represent the qubits to measure which range from 0 to n-1. The sum of the probability of all the measured states should equal to 1.

S-2 Input and output

An example of input is shown below with the instructions defined in Section S1. For simplicity, the input is shown in three columns. Generally, the input consists of three parts: first the configurations of qubits and classical registers, then the body of quantum operations, finally the measurement.

Upload the input script and submit the task to the supercomputer to start to run. If the input is written legally, measurement results will be stored in the output file; otherwise, the error information will be stored in the log.txt file. An example of output is shown below. The first column is the computational basis to measure, and the second one is the probability of the corresponding basis (corresponding to the PMEASURE instruction in the input).

Example: Input

```
%Configure
QINIT 5
CREG 3
%Operate
H 1
S 2
X 2
H 2
CR 1,2,”pi/2”
CR 0,2, ”pi/4”
H 1
CR 0,1,”pi/2”
```

```
S 2
H 0
SWAP 0,2
TOFFOLI 0,1,3
RY 3,"-pi/4"
TOFFOLI 0,1,3
RY 3,"pi/4"
CNOT 0,4
RY 4,"-pi/8"
CNOT 0,4
RY 4,"pi/8"
DAGGER
```

```
X 2
H 2
CR 1,2,”pi/2”
CR 0,2, ”pi/4”
H 1
CR 0,1,”pi/2”
H 0
SWAP 0,2
ENDDAGGER
%Measure
PMEASURE 4,3,0
```

Example: output

```
000: 0.820082
001: 0
010: 0.106694
011: 0
100: 0.0549175
101: 0
110: 0.0183058
111: 0
```
